# Dysregulated Immunity and Immunotherapy after Sepsis

**DOI:** 10.3390/jcm10081742

**Published:** 2021-04-17

**Authors:** Dijoia B. Darden, Lauren S. Kelly, Brittany P. Fenner, Lyle L. Moldawer, Alicia M. Mohr, Philip A. Efron

**Affiliations:** Department of Surgery, College of Medicine, University of Florida, Gainesville, FL 32610, USA; Dijoia.Darden@surgery.ufl.edu (D.B.D.); Lauren.Kelly@surgery.ufl.edu (L.S.K.); Brittany.Fenner@surgery.ufl.edu (B.P.F.); Lyle.Moldawer@surgery.ufl.edu (L.L.M.); Alicia.Mohr@surgery.ufl.edu (A.M.M.)

**Keywords:** PICS, CCI, MDSC, inflammation, immunosuppression, sepsis

## Abstract

Implementation of protocolized surveillance, diagnosis, and management of septic patients, and of surgical sepsis patients in particular, is shown to result in significantly increased numbers of patients surviving their initial hospitalization. Currently, most surgical sepsis patients will rapidly recover from sepsis; however, many patients will not rapidly recover, but instead will go on to develop chronic critical illness (CCI) and experience dismal long-term outcomes. The elderly and comorbid patient is highly susceptible to death or CCI after sepsis. Here, we review aspects of the Persistent Inflammation, Immunosuppression, and Catabolism Syndrome (PICS) endotype to explain the underlying pathobiology of a dysregulated immune system in sepsis survivors who develop CCI; then, we explore targets for immunomodulatory therapy.

## 1. Introduction

**Figure 1:**
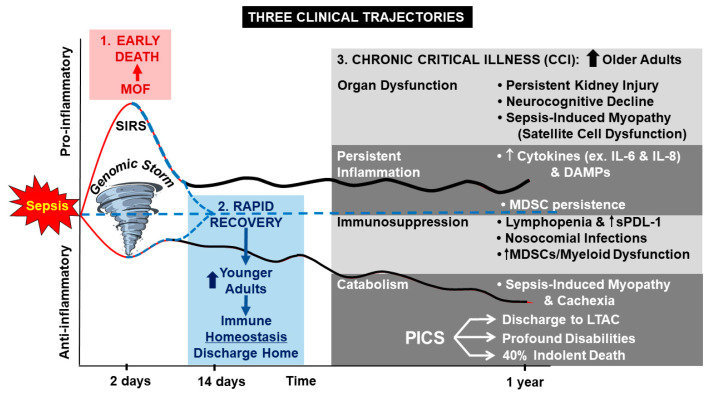
Proposed hypothesis for Persistent Inflammation, Immunosuppression, and Catabolism Syndrome (PICS) in sepsis survivors. Abbreviations: MDSC—myeloid-derived suppressor cell; DAMP—damage-associated molecular protein; LTAC—long-term acute care facility.

Sepsis remains one of the leading causes of death in the United States [1]. Implementation of the 2004 and 2011 Surviving Sepsis Campaign guidelines, as well as of the Centers for Medicare & Medicaid Services guidelines for sepsis management [2], and an increase in systemic and protocolized surveillance, diagnosis, and management of sepsis, all together led to a significant decrease in early death and an increase in hospital survival [3,4]. However, this increased initial survival after sepsis is not yet translated to similarly improved long-term outcomes nor full recovery [5,6]. Specifically, following surgical sepsis, three common clinical trajectories are described: early death, rapid recovery, or development of chronic critical illness (CCI) (Figure 1). Historically, many patients would develop fulminant multiple organ failure from systemic inflammatory response syndrome, leading to early death. Currently, less than 10% of surgical sepsis patients succumb to early death [5]; the majority of patients survive sepsis and either rapidly recover or develop CCI [5,7]. Unfortunately, almost one-third of surgical sepsis patients develop CCI and have dismal long-term outcomes [5].

CCI is defined in several ways, but an accepted definition in the literature is an individual with a prolonged intensive care unit stay (>14 days) and persistent organ dysfunction ranging from low-grade organ insufficiency to chronic organ failure [8,9]. Patients who develop CCI are more likely to be older males with a greater number of medical comorbidities [10,11]. Importantly, patients who develop CCI continue to consume vast resources long after hospital discharge [12]. Additionally, CCI patients have an increased number of secondary infections [13,14] and have poor long-term outcomes that include functional and neurocognitive impairments, increased muscle wasting, and higher 30-day and 1-year mortality [5,9,10,15]. However, the underlying pathobiology of CCI remains unclear.

In a 2012 review, the University of Florida Sepsis and Critical Illness Research Center, under the leadership of Dr. Frederick Moore, defined the Persistent Inflammation, Immunosuppression, and Catabolism Syndrome (PICS) as the clinical endotype underlying the CCI phenotype [16]. PICS is not specific to a critical illness and is seen in a number of conditions including sepsis, trauma, advanced cancer, and chronic inflammatory diseases [16,17]. A significant subset of CCI patients progress to PICS, experiencing ongoing inflammation (e.g., neutrophilia) and immunosuppression (e.g., lymphopenia) that are associated with a sustained acute phase response (e.g., high C-reactive protein and low prealbumin) and persistent whole body protein catabolism [8,18,19]. Clinically, PICS patients (as stated above) suffer from recurrent nosocomial infections and poor wound healing, and frequently develop decubitus ulcers. Despite aggressive nutritional intervention, there is a constant loss of lean body mass accompanied by a proportional reduction in both functional status and wound healing potential [14]. Patients with PICS are commonly discharged to long-term acute care facilities where they face failure to rehabilitate, sepsis recidivism requiring re-hospitalization, and ultimately sufferance of an indolent death [20].

The Persistent Inflammation, Immunosuppression, and Catabolism Syndrome represents a testable hypothesis to elucidate what drives the development of CCI and its morbidities, including persistent immunologic dysfunction, lack of organ recovery, and functional deficit. All of these factors contribute to poor long-term outcomes after severe pro-inflammatory insults such as trauma, sepsis, pancreatitis, or burn injury [21]. Importantly, the PICS endotype offers insight into the dysregulated immunity and dysfunctional emergency myelopoietic response seen in CCI patients after sepsis [7,8,22]. Sepsis is a complex disease process in which outcomes are affected by both the early and late inflammatory response [23,24]. In this review, we will focus on the pathobiologic processes that contribute to the post-sepsis PICS endotype.

## 2. Persistent Inflammation

During acute sepsis, the progression from sepsis to CCI/PICS is mediated by widespread innate immune activation in the early inflammatory response [25]. In sepsis survivors who develop CCI, there remains a persistent elevation in a number of inflammatory markers at least 28 days after sepsis onset, and potentially much longer [26]. The body’s response to sepsis or severe injury begins with the recognition of alarmins derived from either microbial products (pathogen-associate molecular patterns; PAMPs) or tissue injury (damage-associated molecular patterns; DAMPs) [23]. Alarmins represent an array of ligands for highly-conserved pattern recognition receptors (PRRs) that detect exogenous microbial components or host danger signals [23]. These alarmins were previously identified as major mediators of persistent inflammation in CCI after sepsis.

Major classes of PRRs include Toll-like receptors (TLRs), C-type lectin receptors, nucleotide-binding oligomerization domain-like receptors, retinoic-acid-inducible gene-I-like receptors, and receptors for advanced glycation end products (RAGE) [27]. PRRs are comprised of both cell membrane and cytoplasmic receptors that can collectively recognize microbial peptidoglycans, lipopolysaccharides, glucans, phospholipids, high mobility group box (HMGB) proteins [28], S100 family of proteins, nucleic acids [29,30,31], glycoproteins and glycolipids [32], flagellin [33], glycosylated end products [28,34], and oxidation/nitrosylation products associated with cellular injury [27,35,36,37]. Upon recognition of these molecular patterns, PRRs initiate downstream signaling events that induce a host-protective response [36].

In the case of sepsis, alarmins are mostly derived from foreign pathogen materials or PAMPs [27,36]. Among the critically ill, PAMPs are released during the initial sepsis event and in secondary nosocomial infections or reactivated viral infections. PAMPs from these microbes induce a rapid activation of effector cells [36]. PAMPs initiate dendritic cell maturation to promote antigen processing, MHC expression, and migration to lymph nodes to activate T cells [36,38]. In one study of critically ill patients, viral DNA from either CMV, EBV, HHV-6, or TTV was identified in the blood of nearly 87% of critically ill septic patients, compared with <15% for non-septic critically ill patients and healthy controls [39]. Of note, nearly 43% of the septic patients that were examined tested positive for two or more viruses [39].

The other source of alarmins comes from endogenous nucleic acids, proteins, and metabolites released from cellular stress, death at sites of injury, or active secretion from immune cells [40,41]. DAMPs include HMGB1, heat shock proteins, nuclear and mitochondrial DNA and structural peptides such as hyaluron, cellular intermediates such as adenosine, and the cytokines, interleukin (IL)-1α and IL-33 [41,42,43]. Multiple DAMPs, including nuclear DNA, RAGE, and S100, are significantly elevated in sepsis survivors throughout their entire hospitalization [44,45,46,47]. DAMPs are initially released from acutely inflamed and injured tissue secondary to initial septic insult. It is thought that DAMPs are also chronically released from ongoing oxidant and mitochondrial injury in the kidneys, lungs, and intestines of patients with CCI, contributing to a continued low-grade inflammation in these patients after sepsis [7].

**Figure 2:**
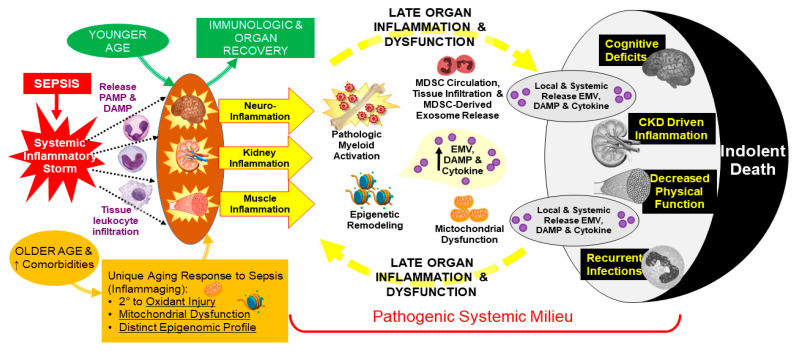
Pathophysiology of Chronic Critical Illness (CCI) and Persistent Inflammation, Immunosuppression, and Catabolism Syndrome (PICS). Abbreviations: DAMP—damage-associated molecular protein, EMV—exosome and microvesicle.

The persistent low-grade inflammation seen in CCI/PICS after sepsis, similar to the chronic low-grade inflammation experienced in cancer and other inflammatory disorders, results in a constant cycle of inflammation-induced organ injury and injury-induced inflammation [17]. The chronic inflammation in PICS induces increased mitochondrial production of reactive oxygen species [48,49,50]. This oxidative stress leads to mitochondrial dysfunction, oxidative damage, and cellular metabolism energy deficits, which further aggravates inflammation and cell death pathways, contributing to low-grade persistent inflammation and organ injury [51]. Continued kidney and muscle damage from inflammation results in further release of DAMPs from these tissues, which further perpetuates this cycle of inflammation [52]. The sepsis-induced chronic inflammation in the brain, termed sepsis-associated encephalopathy, leads to declines in cognitive function and related reductions in quality of life among survivors of sepsis [53,54]. This is especially detrimental in the older patient who already suffers from “inflammaging”, the gradual deterioration of host-protective immunity associated with natural aging [55]. This vicious cycle of inflammation leads to more organ failure and eventually death (Figure 2).

## 3. Persistent Immunosuppression

Immunosuppression after sepsis is reflected in a reduced acute lymphocyte count (ALC) and monocyte membrane (m) HLA-DR expression alongside an increase in soluble programmed cell death ligand-1 (PD-L1), which persists for weeks after sepsis [56]. Low ALC remains suppressed in CCI patients, while it returns to baseline in patients that rapidly recover [56]. Additionally, mHLA-DR is decreased in those that developed secondary infections [57] and is an independent predictor of nosocomial infections [58]. Macrophage and T-lymphocyte dysfunction are important contributors to the PICS-associated immunosuppression [59]. In late sepsis, a state of immune “paralysis” develops as bacterial clearance, cytokine release, and capacity for antigen presentation all decline [59,60]. Additionally, there is a relative lymphocyte “exhaustion” characterized by dysfunctional T cell differentiation and decreased ability to respond to new or continued antigen presentation [61,62,63].

This ongoing immune dysfunction, seen mostly among sepsis-induced CCI survivors, results in increased vulnerability to secondary infections after sepsis. There is a 25–32% readmission rate among all sepsis survivors, with 52–66% of these admissions being for recurrent sepsis [14]. Furthermore, sepsis survivors with CCI experience increased secondary and nosocomial infections at a rate two and a half times greater when compared to patients who rapidly recovered, with a 60% readmission rate for those with CCI [64]. Mortality approaches 40% at six months for patients with CCI, largely due to sepsis recidivism [65,66,67].

## 4. Persistent Catabolism

Sepsis is associated with increased protein breakdown and suppressed protein synthesis, resulting in increased muscle catabolism and a release of muscle-derived DAMPs. Patients with CCI experience a prolonged state of this catabolism with muscle wasting and cachexia that contribute to poor long-term functional outcomes. This breakdown occurs despite enteral supplementation and may require increased protein enrichment with specific additives [15].

There is a plethora of evidence that supports prolonged muscle wasting in late sepsis [15,68,69]. Brakenridge et al. [70] demonstrated that glucagon-like peptide 1, a biomarker of catabolism, is elevated at 24 h and remains elevated at 21 days in sepsis survivors with CCI. Unfortunately, the exact mechanism(s) of sepsis-induced catabolism is not known. There is some evidence that the sustained protein catabolism seen in CCI is partially due to the self-perpetuating inflammation and mitochondrial oxidant injury that leads to the continued release of DAMPs driving inflammation and leading to continued breakdown of skeletal muscle. For example, circulating mitochondrial DNA (mtDNA) present in aging and muscle wasting disorders is also seen in sepsis [71]. DAMPs such as mtDNA, HMGB1, and mitochondrial transcription factor A are increased in systemic circulation during periods of catabolism and continue to drive persistent inflammation [72,73].

## 5. Dysregulated Myelopoiesis

Infections and sepsis also induce emergency myelopoiesis [74,75]. Acute infection initiates mobilization of mature myeloid cells that leads to depletion of bone marrow stores and subsequent release of more immature populations [74]. Early stem cells and multipotent progenitor cells all express PRRs, respond to alarmins, and undergo expansion in response to sepsis. Preferential myeloid progenitor expansion is mediated by inflammatory cytokines and chemokines, including the granulocyte-macrophage-colony stimulating factor (GM-CSF) and the granulocyte-colony stimulating factor (G-CSF) [74]. In a murine model of chronic sepsis, 95% of murine bone marrow cells were noted to be myeloid cells at seven days, with the majority of these cells being phenotypically immature and functionally immunosuppressive [76].

Myeloid-derived suppressor cells (MDSCs) were extensively studied in chronic critical illness of cancer patients and more recently emerged as important immune cells in early and late sepsis [77,78,79]. In the early phases of sepsis, strong signals from DAMPs, PAMPs, and various cytokines and chemokines stimulate rapid mobilization of differentiated monocytes and granulocytes from the bone marrow via classic myelopoiesis [80]. However with persistent weak signaling seen in chronic inflammatory states, such as late sepsis or cancer, there is a shift towards the mobilization and pathologic activation of immature myeloid populations [81]. MDSCs are a heterogeneous group of immature myeloid cells that undergo expansion during emergency myelopoiesis in an attempt to preserve host innate immunity in these pathologic conditions [82]. MDSCs consist of two major groups of cells: polymorphnuclear (PMN-MDSC) and monocytic (M-MDSC) [52]. Phenotypically, PMN-MDSCs are defined as CD11b^+^CD14^−^CD15^+^CD66b^+^LOX-1^+^ with low side scatter (SSC), and M-MDSCs are defined as CD14^+^CD15^−^HLA-DR^−/lo^ with low SSC [83]. Functional characterization of these cells is contingent upon their immunosuppressive functions, i.e., their ability to suppress lymphocyte proliferation and cytokine production [82]. Studies demonstrated that circulating MDSCs are persistently elevated out to 28 days after severe sepsis and septic shock, and are associated with an increase in secondary infections, increased ICU stay, and poor functional status [74,84,85]. In a study of surgical sepsis survivors, MDSCs remained significantly elevated for six weeks post-infection; those same MDSCs only demonstrated suppressive properties at and beyond 14 days post-sepsis [86]. As patients continue to experience unresolved inflammation, there is continued expansion and an eventual pathologic activation of MDSCs [52]. Although the initial expansion of MDSCs may be beneficial by potentiating the early innate immune response and pathogen surveillance, persistent MDSC expansion can be detrimental, as it both propagates persistent inflammation and dampens the adaptive immune response via T cell suppression [82,85,87,88].

## 6. Immunotherapy

Despite the recent decreases in sepsis mortality thanks to earlier recognition and standardized management, the poor long-term outcomes experienced by sepsis survivors with CCI lend evidence to a continued need to investigate agents for use in preventing undesirable sepsis outcomes. At its core, CCI/PICS is an appropriate early inflammatory response that goes awry when it becomes persistent and unabated. The goal of most interventional studies is to bring about a return to immune homeostasis through leukocyte growth factors that suppress MDSCs, promote restoration of normal lymphocyte numbers and function, and/or restore mature functional myeloid populations (Table 1). Leukocyte growth factors such as GM-CSF and G-CSF are one such focus. In one clinical trial, recombinant GM-CSF therapy in immunosuppressed pediatric patients with sepsis restored tumor necrosis factor production in lymphocytes and reduced nosocomial infections to zero in the treatment group [89]. Two other randomized clinical trials involving recombinant G-CSF in severe sepsis and community acquired pneumonia demonstrated an increase in total leukocyte counts in patients receiving the experimental therapy; however, there were no significant improvements in 28-day mortality [90,91]. Similarly, a meta-analysis of 12 clinical trials using recombinant G-CSF and GM-CSF as sepsis treatments in humans found a significant improvement in the rate of infection clearance, but failed to demonstrate any significant improvements in mortality [92]. We attribute this to a failure to address the expansion of pathologically activated MDSCs stimulated by these colony-stimulating factors.

Of note, none of these trials focused on long-term outcomes after 28 days. The use of standard 28-day mortality rates as an endpoint can be misleading and fail to capture the delayed and protracted course of sepsis-related deaths after hospital discharge accurately [93,94]. Though further studies are warranted to assess effects on long-term outcomes, their documented role in reducing infections suggests that there may yet be a role for G-CSF/GM-CSF as one element of a multidrug treatment in combination with other immunotherapies.

There also were studies aimed at directly impacting T cell immunity in sepsis [95,96]. IL-7 is a hematopoietic cytokine that promotes B and T cell development, proliferation and enhancement of T-cell activation, and mobilization to sites of injury [97,98,99]. IL-7 is noted to increase CD4^+^ and CD8^+^ T cell numbers in murine sepsis by upregulating the expression of the anti-apoptosis regulator B-cell lymphoma 2 protein, which is associated with improved survival [99,100]. IL-7 improves ex vivo lymphocyte function [97], and administration of IL-7 enhances T cell receptor diversity in humans, which is typically reduced in sepsis [96,101]. Treatment of sepsis with IL-7 is promising–it was shown to be well-tolerated in clinical trials with no severe toxicities [102]. Furthermore, IL-7 administration more than doubles the levels of circulating CD4^+^ and CD8^+^ T cells in HIV and cancer patients, and preferentially promotes effector T cells instead of regulatory T cells [103,104,105,106]. A follow-up trial is underway to study the effect of IL-7 in restoration of absolute lymphocyte counts in septic patients (NCT03821038).

Interferon gamma (IFN-γ) was also the target of immunomodulatory therapies in inflammatory disease. IFN-γ is important for immune activation against viral, bacterial, and protozoal infections [107]. IFN-γ production is typically suppressed during sepsis in rodents and humans [99,108,109]. However, studies show that the restoration of IFN-γ production improves survival in murine sepsis [99,110]. Treatment with recombinant IFN-γ was associated with increased mHLA-DR expression on monocytes in septic patients and improved monocyte function [111,112,113]. In one randomized controlled trial, IFN-γ treatment decreased infection-related and overall mortality in severely injured trauma patients [114]. IFN-associated genes were suppressed in trauma patients with complicated outcomes, which highlights a potential subgroup for recombinant IFN-γ therapy or IFN-stimulating agents [115,116]. IFN-γ could be a potential immunomodulatory therapy during sepsis. However, as Patil points out, this benefit may ultimately be limited to those with already downregulated mHLA-DR [117]. A clinical trial is underway to examine the effects of IFN-γ on immune function in septic patients (NCT01649921).

An alternative approach is to target the immunosuppressive properties of mature leukocyte populations. Inhibitors of negative co-stimulatory pathways and immune checkpoint inhibitors emerged as potential targets for immunomodulation in sepsis. PD-1 blockade showed promising results in cancer therapeutics [118]. As a result, the PD-1/PD-L1 pathway is an ongoing target for the treatment of sepsis. PD-L1 and its receptor, PD-1, serve as a checkpoint inhibitor responsible for limiting CD8^+^ T cell proliferation and accumulation in lymph nodes. PD-1 is upregulated on CD4^+^ and CD8^+^ T cells in states of infection and inflammation [119,120]. High levels of PD-1 are associated with elevated secondary infection and mortality rates, and limited T cell proliferation among critically ill patients [119]. PD-1 knockout mice have improved effector T cell proliferation and faster adenovirus clearance [121]. In vitro PD-1/PD-L1 blockade decreases T cell apoptosis and IL-10 release, and improves the function of neutrophil and monocytes from septic mice and humans [122,123]. In vivo, it appears to restore impaired CD8^+^ T cell function, leading to improved cytokine release and decreased viral loads (even in CD4-deficient models), with this improved functionality persisting for weeks following the transient blockade [124]. In pre-clinical mouse models of sepsis, blockade of other proteins in the PD-1/PD-L1 pathway significantly improved survival as well [110]. Additionally, inhibition of the PD-1/PD-L1 pathway prevented lymphocyte depletion and apoptosis, and improved survival after CLP in mice [125,126]. However, just as with other clinical trials in sepsis, there yet is no study to assess the effects of PD-1/PD-L1 inhibition on long-term outcomes beyond 30 days.

**Table 1:** Summary of select immunotherapy studies in sepsis or immunodeficiency.

Intervention	Result	Ref
GM-CSF	Restoration of monocytic immunocompetence. Shortened time of mechanical ventilation and hospital stay.	[89]
G-CSF	Increased total leukocyte counts. No difference in mortality rates or complications in sepsis patients.	[90]
G-CSF & GM-CSF	Improved infection clearance, but no difference in mortality rates in sepsis patients.	[92]
IL-7	Improved lymphocyte counts (CD4^+^ and CD8^+^ immune effector cells) in sepsis patients.	[102]
IL-7	Increased CD4/CD8 T cells in HIV patients.	[104]
IL-7	Increased CD4/CD8 T cells in patients with lymphopenia.	[105]
IFN-γ	Increased HLA-DR expression and decrease in natural killer cells in patients with sepsis.	[111]
IFN-γ	Decreased infection related mortality and overall mortality in trauma, but no difference in infection rates in trauma patients.	[114]
PD-1/PD-L1 blockade	Ex-vivo restoration of function in neutrophils, monocytes, T cells, and NK cells in whole blood from septic patients.	[122]
PD-1/PD-L1 blockade	In-vitro decreased T-cell apoptosis, potentiated monocytic LPS-induced TNF-α and IL-6 production from sepsis patients.	[123]

Newly emerging interest in the role of MDSCs in sepsis triggered studies to target these cells for therapy. Although MDSCs were demonstrated to improve bacterial clearance, persistent activation also results in the failure to resolve acute inflammation and immunosuppression, ultimately leading to increased mortality [127]. Attenuating or modifying MDSC activation, expansion, and migration may be another approach to the treatment of sepsis [128]. A multitude of clinical efforts are underway to target MDSCs’ number and function in cancer [129]. The implications of such approaches after sepsis are less clear-cut; however, many immunomodulatory targets to attenuate MDSC immunosuppression already emerged. Studies limiting MDSC expansion and functionality using gemcitabine-treated or CCAAT enhancer binding protein beta-knockout mice yielded conflicting results. After burn injury, gemcitabine treatment successfully resulted in a decrease in MDSCs and increased survival following a lethal dose of LPS, but conferred a decreased survival to *Pseudomonas aeruginosa* infection [130]. In another study, deficiency in MDSC signaling pathways caused persistent elevation in inflammatory cytokines and worsened survival, which all improved with the reintroduction of MDSCs [131]. Though there are no current clinical trials targeting MDSCs in sepsis, future trials should take into account the timing of MDSC modulatory treatment, as the MDSC immunosuppressive function is not seen until day 14 after sepsis [86].

## 7. Conclusions

Thanks in no small part to concerted efforts and global campaigns over the last two decades, patients with sepsis are surviving in greater numbers early in their hospitalization. Unfortunately, many of those who survive do not rapidly recovery and instead experience prolonged intensive care unit stays and persistent organ dysfunction. CCI after sepsis is associated with long-term dismal outcomes out to one year from onset, including poor functional status, recurrent infection, failure to rehabilitate, and increased mortality [5]. The pathobiology of CCI is likely multifactorial but can be partially explained as being driven by the constellation of inflammatory, immunologic, and metabolic dysregulation, collectively defined as PICS. Single therapies that target aspects of PICS have yet to be successful, but long-term adverse outcomes after sepsis may be best attenuated with multimodal therapy. It is clear from clinical trials that a “one-size-fits-all” treatment strategy does not, in fact, fit all. Therefore, the multimodal therapy may require a precise, personalized strategy to be successful in helping patients not only survive sepsis, but also offering them a better chance for good functional recovery.

## Data Availability

Not applicable.
